# Recurrent hypopigmented macules and papules in a young male: An extragenital presentation of guttate lichen sclerosus

**DOI:** 10.1016/j.jdcr.2025.05.047

**Published:** 2025-07-09

**Authors:** Makenna Chapman, Marie Chaghouri, Aislyn Oulee, Ashley N. Elsensohn, Christina N. Kraus, Brigette Lee

**Affiliations:** a University of California Irvine School of Medicine, Irvine, California; b Department of Dermatology, University of California Irvine, Irvine, California; c Department of Dermatology, Loma Linda University, Loma Linda, California

**Keywords:** case reports, dermoscopy, lichen sclerosus

## Case

**Fig 1:**
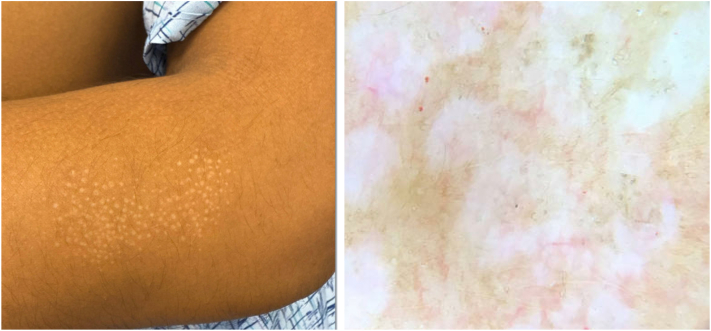
Arm displaying multiple discrete drop-shaped *white*, atrophic, shiny papules.

**Fig 2:**
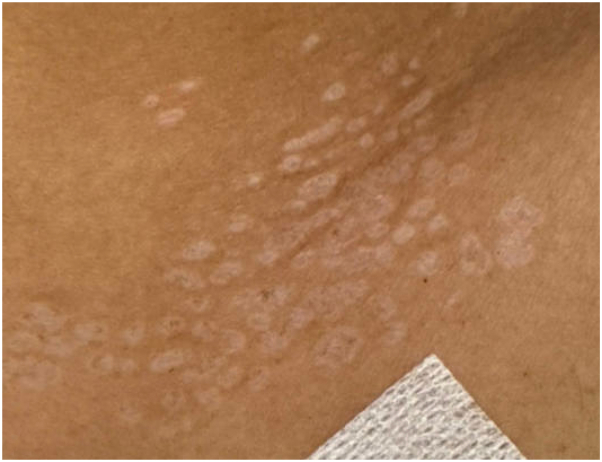
Neck with multiple *white* to *pink* atrophic papules.

**Fig 3:**
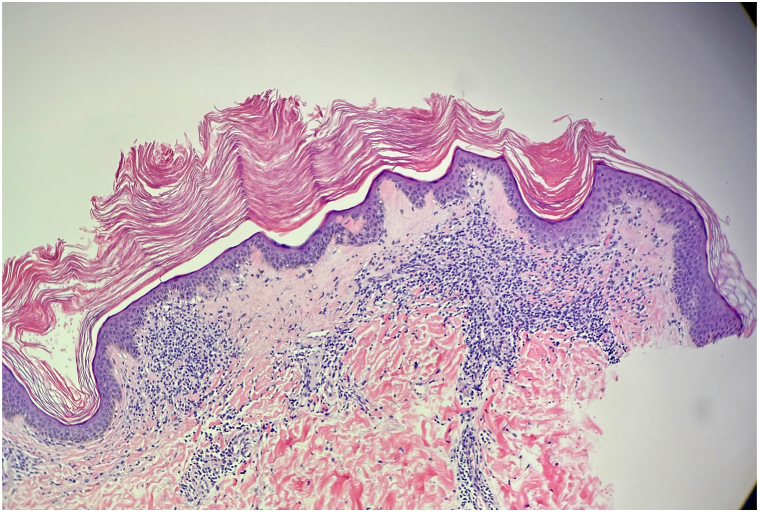
Hematoxylin and eosin (H&E) 10× hyperkeratosis overlying a lichenoid interface dermatitis with collagen homogenization in the papillary and superficial reticular dermis.

A 26-year-old male presented with a history of recurrent, asymptomatic lesions involving the right flank and neck (Figs 1, *A* and *B*, 2 and 3). Lesions appeared every few months, lasted weeks and resolved spontaneously. While nonpruritic, they occasionally became pink to red. Examination revealed multiple small white to pink atrophic macules and papules. A punch biopsy of the right flank revealed orthokeratosis overlying a lichenoid interface dermatitis with areas of collagen homogenization in the papillary dermis (Fig 3).

**Question: What is the most likely diagnosis and the key dermoscopic features?**

**A.** Atrophic lichen planus – White wickham striae with dotted vessels and background erythema
**B.** Guttate lichen sclerosus (GLS) – Homogenous structureless white to pink areas with irregular linear vessels and comedone-like openings
**C.** Inflammatory vitiligo – Depigmented macules with erythema at margins and perifollicular pigmentation
**D.** Malignant atrophic papulosis – Erythema surrounding porcelain white center containing hairpin vessels
**E.** Guttate morphea – White fibrotic beams with linear telangiectasias

## Discussion

**Correct answer: B. GLS** – Homogenous structureless white to pink areas with irregular linear vessels and comedone-like openings.

GLS is an uncommon extragenital variant of lichen sclerosus (LS) presenting as small, scattered, drop-like atrophic or shiny white macules and papules. While LS frequently involves the genital region, ∼15% to 20% of cases are extragenital. Diagnosis is primarily clinical, supported by histopathology revealing lichenoid interface dermatitis, dermal collagen homogenization, and epidermal changes, including hyperkeratosis, loss of rete ridges, and follicular plugging. Dermoscopy may aid diagnosis, revealing patchy yellow-white to white-pink structureless areas, linear or dotted vessels, comedone-like openings, and white chrysalis-like structures with surrounding erythema.^1^^,^^2^ Irregular linear vessels have been reported in “early” lesions.^2^

GLS can be differentiated from other lichenoid or sclerotic dermatoses clinically, histologically, and with dermoscopy. Inflammatory vitiligo, which may be guttate, lacks the lichenoid infiltrate and collagen changes seen in LS. Histology shows epidermal melanocyte loss, while dermoscopy reveals depigmented macules with perifollicular pigmententation and marginal erythema.^1^^,^^2^ Atrophic lichen planus may mimic LS clinically with a lichenoid interface dermatitis histologically. However, it lacks dermal collagen changes and exhibits wickham striae under dermoscopy.^1^ Guttate morphea may resemble GLS clinically but is distinguishable dermoscopically by white fibrotic beams.^3^ Histology reveals deeper dermal sclerosis without the lichenoid band and follicular plugging seen in LS.^1^ Malignant atrophic papulosis can resemble GLS but is a thrombo-obliterative vasculopathy with histological wedge-shape pallor in the deep dermis, perivascular lymphocytic infiltrate in the reticular dermis, and vasculopathic features such as fibrin and thrombi. Dermoscopy reveals a central porcelain-white area with surrounding telangiectasias.^1^^,^^4^

Dermoscopy, alongside histology, is useful in evaluating patients with atrophic papules and macules and identifying cases of GLS.^3^ Given the genital predilection of LS, genital exam should also be considered for patients with GLS to support early diagnosis and prevent functional complications. This case highlights an uncommon extragenital LS presentation in a young male without genital involvement, underscoring the importance of recognizing GLS as a distinct variant and including it in the differential for hypopigmented macules and papules.

## Conflicts of interest

Dr Kraus is the recipient of a Dermatology Foundation Career Development Award and has been a consultant for Nuvig Therapeutics, LEO Pharma, and an investigator for Incyte Corporation. Authors Chapman, Chaghouri and Drs Oulee, Elsensohn, and Lee have no conflicts of interest to declare.
